# A Strategy to Battle Coronavirus Disease 2019 in the Hospital: Identify and Use the Power of “Immune Survivors”

**DOI:** 10.31662/jmaj.2020-0039

**Published:** 2020-10-02

**Authors:** Hironori Yamamoto, Kouichi Miura, Koichi Hagiwara, Yuji Morisawa, Alan Kawarai Lefor, Naohiro Sata, Ryozo Nagai

**Affiliations:** 1 Jichi Medical University, Shimotsuke, Japan

**Keywords:** SARS-CoV-2, COVID-19, antibody, immune, nosocomial

## Abstract

The outbreak of the severe acute respiratory syndrome coronavirus 2 (SARS-CoV-2) poses serious health and economic problems worldwide. One of the worst scenarios is the collapse of the medical care system due to nosocomial infections. SARS-CoV-2 quickly spreads in closed spaces, crowded areas, and close physical distances, which frequently occur in Japanese medical facilities. Although we are making efforts to avoid such situations, healthcare workers always face the risk of developing a SARS-CoV-2 infection in the workplace because of proximity. Thus, we need to battle SARS-CoV-2 using a unique strategy.

We propose a novel strategy to eliminate SARS-CoV-2 infections: measurement of antibodies against SARS-CoV-2 and using the power of “immune survivors.” We agree with using standard precautions and early isolation of patients with coronavirus disease 2019 (COVID-19) to block the spread of SARS-CoV-2 infection. However, we face difficulties carrying out these fundamental missions. Now, we focus on “immune survivors.” If healthcare workers acquired the neutralizing antibody against SARS-CoV-2, they are considered “immune survivors” with a low risk of reinfection with SARS-CoV-2. These “immune survivors” can contribute to the care of patients with COVID-19 on the front line. Also, these “immune survivors” can function as an envelope by surrounding COVID-19 patients. As a result, “immune survivors” can eliminate the spread of SARS-CoV-2 in medical facilities as well as in society.

We understand that the concept of “immune survivors” needs further discussion. No information is available on how long or the titer of neutralizing antibody required for protection from infection. We have just started to measure antibody levels against SARS-CoV-2 in healthcare workers in our hospital. This project will provide further information in the battle against the SARS-CoV-2 infection. (Clinical trial registration number: UMIN 000039997)

The severe acute respiratory syndrome coronavirus 2 (SARS-CoV-2) pandemic poses serious health and economic problems throughout the world ^(1)^, including Japan. One of the worst scenarios is the collapse of the medical care system due to the spread of nosocomial infections. Indeed, some medical facilities are temporarily closed due to the spread of SARS-CoV-2 among healthcare workers. Thus, preventing SARS-CoV-2 infections among healthcare workers is an urgent issue in our society.

Although individuals without antibodies are susceptible to SARS-CoV-2, the spread of infection can be suppressed using social distancing. In contrast, SARS-CoV-2 quickly spreads in closed spaces, crowded areas, and close physical distances, which frequently occur in Japanese medical facilities (Figure 1). Although the Japanese government strongly recommends avoiding such situations, healthcare workers always face the risk of developing a SARS-CoV-2 infection in the workplace because of proximity with patients. Thus, we need to battle SARS-CoV-2 using a novel strategy.

**Figure 1.**
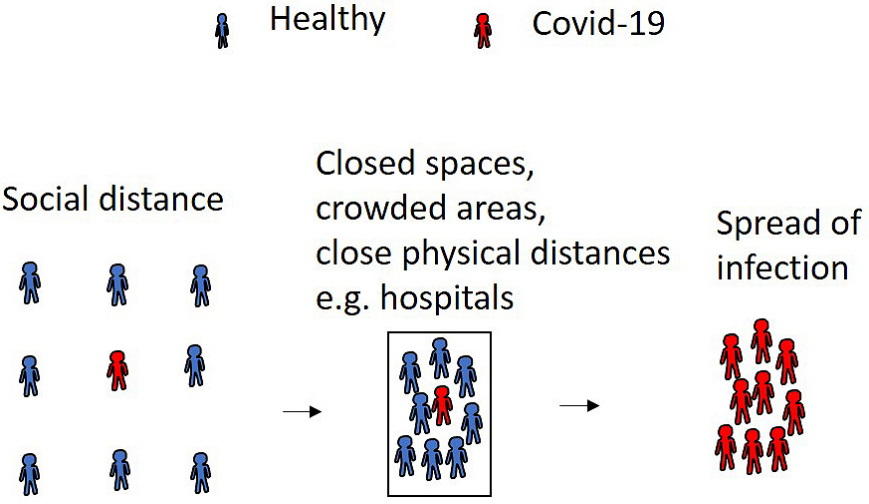
The spread of SARS-CoV-2 infection in closed spaces, crowded areas, and at close physical distances.

The first strategy to prevent this infection in hospitals and clinics is to use standard precautions for SARS-CoV-2, such as personal protective equipment. Currently, it is impossible to test for SARS-CoV-2 infection in all patients who visit medical facilities. As a result, we have to see patients assuming they may be infected. The saga aboard the cruise ship “Diamond Princess” is a good example. A total of 2700 officers from the Japan Self-Defense Forces rescued the cruise ship’s passengers. Surprisingly, none of these servicemen contracted SARS-CoV-2 on their mission. Their action is an excellent example of how we can prevent SARS-CoV-2 infection. Indeed, our co-workers see patients with coronavirus disease 2019 (COVID-19) without contracting SARS-CoV-2. They care for patients with COVID-19 by taking proper precautions.

The second strategy is early isolation of patients with COVID-19. Polymerase chain reaction (PCR) is the gold standard to diagnose COVID-19 infection. However, standard PCR tests require 2-3 hours to complete. Besides, there is an insufficient number of PCR test kits to test all patients suspected to have COVID-19. A rapid detection kit for the SARS-CoV-2 antigen is now available. Although the accuracy of detecting SARS-CoV-2 is likely lower than that of the PCR test, rapid antigen detection kits will be a powerful tool to identify patients with COVID-19 who should be isolated.

We propose a third strategy: measuring antibodies against SARS-CoV-2 and using the power of “immune survivors.” Although the antibodies are not yet well characterized, IgM and IgG antibody levels can be measured in patients with SARS-CoV-2 infection. We focus on the potential of the IgG antibody as a neutralizing antibody. If a healthcare worker acquires the neutralizing antibody against SARS-CoV-2, that healthcare worker is considered an “immune survivor,” who carries a lower risk of infection with SARS-CoV-2. However, the inappropriate deployment of “immune survivors” does not exert the maximum effect to prevent the spread of SARS-CoV-2 (Figure 2A). If these “immune survivors” contribute to the voluntary care of patients with COVID-19 on the front line, these “immune survivors” can function as a protective wall, surrounding patients with COVID-19 (Figure 2B). As a result, the “immune survivors” can terminate the spread of SARS-CoV-2 in hospitals and clinics. This novel strategy can be applied to our community.

**Figure 2.**
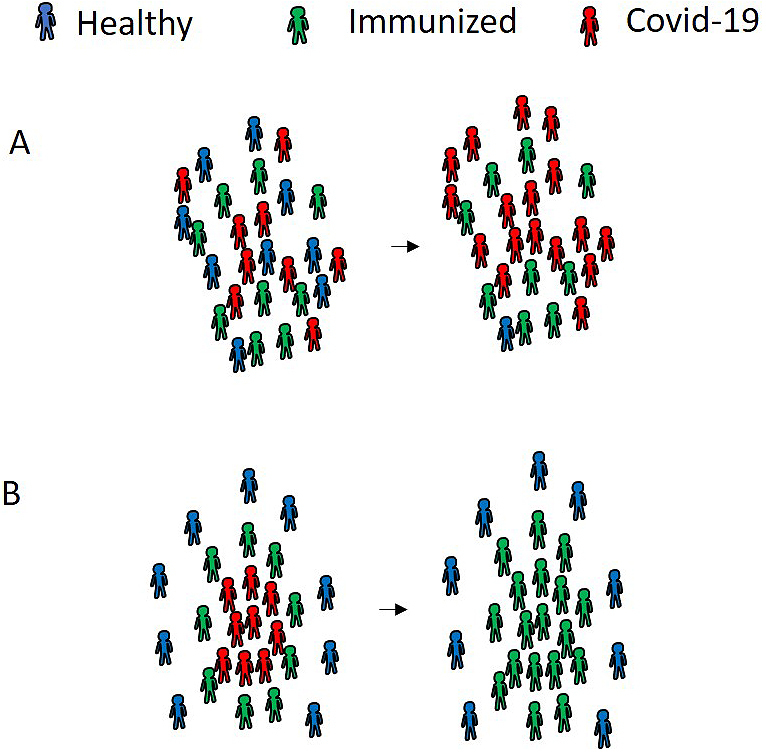
A model to prevent the spread of SARS-CoV-2 using the power of “immune survivors.” The proliferation of SARS-CoV-2 occurs if “immune survivors” do not interrupt the contact between healthy individuals and patients with COVID-19. In contrast, a small number of “immune survivors” can block the spread of the infection if we identify and arrange “immune survivors” and patients with COVID-19 at an earlier point in time.

Identifying the “immune survivors” is crucial to completing our strategy. Two consecutive PCR tests can determine whether patients have been cured of COVID-19. However, relapses among “cured patients” confuses our approach. Measuring both IgM and IgG antibodies may be a useful marker. There were a certain number of patients who were still positive by PCR testing even after their symptoms disappeared. A high titer of IgM antibody was maintained in such patients with prolonged-RNA-shedding ^(2)^. These data suggest that the IgM antibody can be a surrogate marker for PCR testing to identify prolonged-RNA-shedding patients. Thus, results showing IgM negative but IgG positive could be useful for identifying “immune survivors.” Measuring these antibodies may provide an additional benefit for individuals who have a risk of contracting SARS-CoV-2. An unexpected problem associated with this infection is “further social distancing,” namely avoidance and/or discrimination within the community. Indeed, family members of healthcare workers have difficulties obtaining some social services, such as childcare. If the public-at-large understands the concept of “immune survivors,” this problem can be resolved.

Based on our hypothesis, we have already started measuring levels of IgM and IgG antibodies against SARS-CoV-2 among healthcare workers at Jichi Medical University Hospital. The Institutional Review Board of Jichi Medical University approved this clinical research projects (Permission number of Institutional Review Board, A19-200). The study was registered with the UMIN Clinical Trials Registry (https://www.umin.ac.jp/ctr/index-j.htm, Clinical trial registration number: UMIN 000039997). At present, 54 healthcare workers (1.4% of hospital employees) are enrolled in this study and have taken a rapid detection test (Kurabo, Osaka, Japan). Two doctors complained of fever and respiratory symptoms during the study period (PCR tests for SARS-CoV-2 were negative). Two other doctors were in Europe, a pandemic area, in March 2020. Two different doctors saw patients with COVID-19 with enhanced precautions. The preliminary results show that 54 healthcare workers were negative for both IgM and IgG antibodies against SARS-CoV-2. These results indicate that our institution completed the first (precautions) and second (isolation of patients with COVID-19) strategies in a storm of SARS-CoV-2.

We understand that the utility of measuring antibody levels is under debate. It is still unclear whether acquired antibodies will act as neutralizing antibodies. We have no information regarding treatment duration or the IgG titer needed for protection from infection. Tan et al. reported that the titer of IgG antibody was positively correlated with the severity of COVID-19 illness ^(3)^, suggesting that a high titer of IgG does not necessarily guarantee clearance of and/or immunity to the virus. Also, the duration of IgG positivity overlaps with PCR positivity, indicating that the use of a single test (PCR or antibody) has the risk of releasing SARS-CoV-2 to society. However, we believe that the immune response pattern in severely ill patients and the acquisition of immunity after recovery from the disease are different. It is reasonable to assume that those who have recovered with acquired antibodies have immunity from reinfection with an acute viral infection such as COVID-19. Indeed, transfusion of convalescent plasma improved in the clinical status of critically ill patients with COVID-19 ^(4)^, suggesting that antibodies produced in typically recovered patients are neutralizing antibodies.

At present, we have several challenges. The supply of personal protective equipment does not meet our needs. The number of patients who can undergo PCR and antigen testing is limited. As a result, the outcome of the battle against SARS-CoV-2 infection is unpredictable. Theoretically, this infection will be terminated when herd immunity has been achieved; that is, 80% of individuals have acquired the neutralizing antibody. Although vaccination will help the acquisition of herd immunity, it is a dangerous process, while 80% of people acquire the neutralizing antibody with no vaccine and/or other treatments. Using the third strategy we have proposed, identifying both infectious people and immune people and arranging for the immune people to surround the contagious people, the infection can be terminated. This strategy mimics herd immunity with a much smaller percentage of immune acquisition in the community. Currently, there is no demonstration that this hypothesis is correct. Although the actual value of this strategy has not yet been proven, we propose it as an option. The battle against SARS-CoV-2 has just begun. We will continue this project and provide further information regarding this strategy against SARS-CoV-2 infection as this observational study progresses. If the second and third waves of SARS-CoV-2 attack society, we believe that these proposed actions can stop the cycle of SARS-CoV-2 infection in medical facilities as well as in our communities.

## Article Information

### Conflicts of Interest

None

### Author Contributions

Hironori Yamamoto: conception and design of the work, revising the work critically, final approval of the version, agreement to be accountable for all aspects of the work.

Kouichi Miura: the acquisition, analysis, and interpretation of data for the work, drafting the work, final approval of the version, agreement to be accountable for all aspects of the work.

Koichi Hagiwara: interpretation of data for the work, revising the work critically, final approval of the version, agreement to be accountable for all aspects of the work.

Yuji Morisawa: interpretation of data for the work, revising the work critically, final approval of the version, agreement to be accountable for all aspects of the work.

Alan Kawarai Lefor: interpretation of data for the work, revising the work critically, final approval of the version, agreement to be accountable for all aspects of the work.

Naohiro Sata: interpretation of data for the work, revising the work critically, final approval of the version, agreement to be accountable for all aspects of the work.

Ryozo Nagai: interpretation of data for the work, revising the work critically, final approval of the version, agreement to be accountable for all aspects of the work.

### Approval by Institutional Review Board (IRB)

A19-200, Jichi Medical University

### Disclaimer

Koichi Hagiwara is one of the Editors of JMA Journal and on the journal's Editorial Staff. He was not involved in the editorial evaluation or decision to accept this article for publication at all.
